# Synthesis of Ag nanoparticles by *Celery* leaves extract supported on magnetic biochar substrate, as a catalyst for the reduction reactions

**DOI:** 10.1038/s41598-022-18131-w

**Published:** 2022-08-11

**Authors:** Sahar Taheri, Majid M. Heravi, Pourya Mohammadi

**Affiliations:** Department of Chemistry, Faculty of Physics and Chemistry, Alzahra University, Vanak, POBox 1993891176, Tehran, Iran

**Keywords:** Environmental chemistry, Organic chemistry, Chemical synthesis

## Abstract

Green synthesis of a noble metal such as Ag nanoparticles is an enormously developed research area. In this study, a biochar/Fe_3_O_4_–Ag magnetic nanocatalyst was produced via a green path by using Celery stalk as a carbon-based substrate and *Celery* leaf extract as reducing and stabilizing agents to construct Ag nanoparticles. The synthesized nanocatalyst was determined using various techniques, such as UV–Vis spectroscopy, FT-IR spectroscopy, XRD (X-ray diffraction), SEM/EDX spectroscopy (scanning electron microscopy/energy-dispersive X-ray), TEM (transmission electron microscopy), and VSM (vibrating sample magnetometer). To survey the catalytic action of the biochar/Fe_3_O_4_–Ag nanocatalyst, it was used in the reduction reaction of disparate nitroaromatics, aldehydes, and ketones. This catalyst has demonstrated good characteristics in terms of the amount, reusability, recoverability, activity, and structural integrity of the catalyst during the reaction. In addition, biochar/Fe_3_O_4_–Ag could be detached magnetically and recycled multiple times without significantly reducing its catalytic performance.

## Introduction

Biochar is a stable and carbon-rich solid that is produced by the pyrolysis of diverse biomasses, such as wood, manure, or leaves, without the presence of oxygen (or only a very small amount)^1–3^. Additionally, biochar can be acquired from raw feedstock such as plant-based raw materials^4^. Biochar has supreme features such as rich carbon content, wide specific surface area, stable and porous structure, high ion exchange capacity, numerous functional groups, easy access, and low price of raw materials^5–7^. Because of its substantial characteristics, such as large surface area, abundant active functional groups, porosity, and pore volume, it has been used for a variety of applications, including environmental remediation, energy storage, water and wastewater treatment, catalyst support, etc.^8,9^. To improve the physicochemical properties of biochar, different processes, such as modification with acids, alkalis, oxidizing agents, and various activation methods, including amination, hydrothermal synthesis, and magnetization, have been employed^10–12^.

Catalysts are very important for human society since more than 90% of all chemical processes need a catalyst for the targeted generation of various products^13^. In recent years, the benefit of noble metal nanoparticles as heterogeneous catalysts has gained much consideration because of their unparalleled virtues, such as large surface area and high chemical activity^14–16^. Silver nanoparticles, as substantial noble metal nanoparticles, have been most extensively utilized and studied owing to their stability, high conductivity, antibacterial properties, specific virtues of size and shape, and particularly their superb catalytic activity^17–19^. Multiple approaches exist for the synthesis of silver nanoparticles^20^. For instance, silver ions are reduced by chemical^21,22^, photochemical^23^, radiation^24^, electrochemical^25,26^, Langmuir Blodgett^27,28^, and biological techniques^29–32^. The naturally occurring substances for biological synthesis consist of plant extracts, plants, and microorganisms^33–36^. In addition, the green synthesis process is a convenient way to synthesize nanoparticles^37^ because it is not only a one-step and eco-friendly method of synthesis but also reduces the consumption or production of hazardous materials to human health and the environment^38–42^.

Recently, the utilization of magnetic nanoparticles (MNPs) as a beneficial material has increased significantly owing to their specific virtues, such as high specific surface area, superparamagnetic properties, small size, nontoxicity, and the large surface-to-volume ratio^43,44^. The accumulation of nanoparticles during the catalytic reaction is one of their impediments^45^. Segregation of nanoparticles from the reaction medium using ordinary methods such as filtration and centrifugation is tough. Therefore, magnetic nanoparticles allow easygoing dissociation from the reaction mixture via an external magnet without the need for further processes that not only omit the necessity of cumbersome centrifugation and filtration procedures but also diminish energy expenditure and catalyst wastage and preserve time in attainment catalyst recovery^46–49^.

Reduction reactions are one of the most substantial and beneficial chemical reactions in the synthesis of organic compounds^50^. Aromatic nitro compounds are widely utilized in different processes. When these compounds are released into the environment, they cause severe injuries to humans, plants, animals, and the whole biological system. From this point of view, the constitution of amines through the reduction of nitro compounds represents a fundamental evolution in organic chemistry^51,52^. Amines, especially aniline and its derivatives, which are indispensable for the construction of pesticides, pigments, polymers, dye intermediates, and pharmaceutics, play a momentous role in the organic chemical industry^53–55^. On the other hand, the reduction of aldehydes and ketones is significant due to the far-reaching utilization of alcohol in pharmaceuticals, agrochemicals, and the preparation of solvents^56^. From petroleum products, we will not obtain a vast range of alcohols, but performing aldehyde and ketone reduction reactions will result in the provision of various alcohols^57^. The tense issue in the synthesis of amines and alcohols is to select the most efficient heterogeneous catalyst to acquire the best catalytic activity, reusability, and persistence. For this purpose, many studies have been accomplished on the reduction of nitro, aldehyde, and ketone compounds in the attendance of diverse heterogeneous catalysts^58–62^. For instance, various solid supports, such as TiO_2_^63^, carbon-based materials (biochar, graphene)^64^, and Fe_3_O_4_, have been used to stabilize metal nanoparticles and fabricate heterogeneous nanocatalysts^65,66^.

Herein, we have reported a green process to provide a novel nanoscale silver particle catalyst supported on a magnetic carbon-based biochar substrate. For the synthesis of the biochar/Fe_3_O_4_–Ag nanocatalyst, initially, a carbon-based biochar substrate was constructed using a celery plant, and then a biochar/Fe_3_O_4_ nanocomposite was prepared in the presence of magnetic nanoparticles. Finally, silver nanoparticles were synthesized utilizing celery leaf extract and immobilized on a magnetic biochar/Fe_3_O_4_ substrate. Therefore, we synthesized a biochar/Fe_3_O_4_–Ag nanocatalyst and exploited it in the reduction of nitroaromatic, aldehyde, and ketone compounds using NaBH_4_ as the reducing agent and H_2_O as the green solvent. In general, the green synthesized nanocatalyst was manufactured using green precursors, solvents, and methods.

## Experimental

### Materials

Fresh Celery, ferric chloride hexahydrate (FeCl_3_·6H_2_O, 97%), ferrous chloride tetrahydrate (FeCl_2_·4H_2_O, 98%), ammonium hydroxide solution (NH_4_OH, 25%), and silver nitrate (AgNO_3_, 99%) were procured from Sigma-Aldrich. Aldehyde and ketone derivatives were purchased from Sigma and Merck.

The leaves were purchased from a local shop in Tehran. The plant we used in this work is a plant that is found in abundance in local shops and is not wild and endangered. This study complies with relevant institutional, national, and international guidelines and legislation.

### Synthesis of biochar/Fe_3_O_4_–Ag nanocatalyst

#### Synthesis of biochar

To prepare the biochar carbon substrate initially, 250 g of celery stalk was rinsed and desiccated at 60 °C for 24 h and then powdered. The green powder (4 g) with distilled water (65 mL) was poured into a 100 mL autoclave. Approximately, the autoclave was warmed up at 180 °C for 24 h and then chilled naturally to ambient temperature. The solid product was separated by centrifugation, washed, and dried. The final black powder was biochar.

#### Synthesis of biochar/Fe_3_O_4_

Generally, 0.5 g of biochar is dispersed to 120 mL of distilled water. Then, 0.5 g of FeCl_2_.4H_2_O and 1.37 g of FeCl_3_·6H_2_O were added to the above mixture and warmed at 35 °C for 3 h. Subsequently, the temperature was up to 60 °C, and 10 mL of NH_4_OH was added drop-wise to the above mixture. Then, the mixture was stirred for an additional 60 min. After cooling to room temperature, the product was separated using an external magnet, washed multiple times with distilled water (H_2_O), and dried at ambient temperature.

### Preparation of leaf extract

One hundred grams of green celery leaves were cut and thoroughly rinsed many times with distilled water (H_2_O) to eliminate mist particles. Afterward, the green leaves (50 g) were extracted by using 300 mL of distilled water at 100 °C for 6 h, after which they were permitted to become cold on their own. Finally, the celery leaf extract solution was filtered and dried at 55 °C. The procurement of leaf extract is shown in Fig. S1.

### Green synthesis of Ag nanoparticles

In a usual reaction process, 2 mL of the leaf extract of 0.25% M was added dropwise to 5 mL of 1.5 mM aqueous AgNO_3_ solution and stirred at 55 °C. The Ag nanoparticles were made from the reduction of silver ions over approximately 3 h. By UV–Vis (UV–Vis) spectroscopy, the reaction was controlled. The color change from light green to brown confirms the reduction of Ag^+^ to Ag^0^.

### Synthesis of biochar/Fe_3_O_4_–Ag

To procure the nanocatalyst, biochar/Fe_3_O_4_ (0.15 g) was added to 20 mL of distilled water (H_2_O) and stirred for 30 min. Afterward, 50 mL of AgNO_3_ (2 mM) solution was appended into the blend and stirred at room temperature for 5 h. Thereafter, the temperature of the reaction was up to 65 °C, and it was stirred for another 30 min. Afterward, 10 mL of aqueous extract (0.25% M) was added to this mixture and stirred for 3 h at 65 °C. The constructed product was detached by an external magnet, washed with distilled water (H_2_O) a couple of times, and desiccated at ambient temperature. The process of biochar/Fe_3_O_4_–Ag nanocatalyst synthesis is shown in Fig. S2.

### Reduction of nitroaromatic compounds catalyzed by biochar/Fe_3_O_4_–Ag

Catalytic reduction reactions of nitro compounds were performed in an aqueous solution at 50 °C in the presence of NaBH_4_ as the reducing agent. In a typical way, nitroaromatic compounds (0.5 mmol) and H_2_O (3.0 mL) were mixed into a 10 mL round-bottom flask and stirred at the desired temperature_._ Then, biochar/Fe_3_O_4_–Ag nanocatalyst (10 mg) and NaBH_4_ (3 mmol) were added, and the final mixture was stirred for a suitable time. The progression of the reaction was controlled by applying TLC (normal hexane–ethyl acetate as solvent). After the finishing of the reduction reaction, the biochar/Fe_3_O_4_–Ag nanocatalyst was segregated by an outer magnet, rinsed with H_2_O and ethanol, and dried to be applied for the next cycle. Finally, to provide pure products, the obtained products were recrystallized from ethanol.

### Reduction of aldehyde and ketone compounds catalyzed by biochar/Fe_3_O_4_–Ag

In a catalytic process, the reduction reactions of aldehyde and ketone compounds were performed. In a usual way, a mixture of 0.5 mmol aldehyde and ketone compounds and 2 mL of water as a solvent was added into a round bottom flask and stirred for 10 min at ambient temperature. Afterward, 5 mg of biochar/Fe_3_O_4_–Ag catalyst and NaBH_4_ (3 mmol) were subjoined into the above mixture, and the whole combination was stirred for an adequate time. The reaction was monitored by TLC (normal hexane–ethyl acetate as the solvent, 2:8). After termination of the reaction, the nanocatalyst was detached by an outer magnet, rinsed with ethanol and H_2_O, and dried to be used in the next cycle. Finally, the product was extracted and purified.

## Results

### FT-IR spectroscopy

To study the structure of the presented nanocatalyst in more detail and characterize the functional groups, the FT-IR spectra of the nanocatalyst fabrication steps of (a) biochar, (b) biochar-Fe_3_O_4_, and (c) biochar/Fe_3_O_4_–Ag were examined, and the results are shown in Fig. S3. The bands present at 3300–3500 cm^−1^ are linked to the OH stretching vibration mode in Fig. S3a. The uptake bands at 2852 cm^−1^ and 2923 cm^−1^ were caused by the stretching vibration of the C–H bond. The bonds observed at 1733 cm^−1^, 1650 cm^−1^, and 1061 cm^−1^ were devoted to the presence of stretching vibrations of the carbonyl group (C=O), C=C, and C–O bonds, respectively. In addition, the broadband at 700–800 cm^−1^ corresponded to the out-of-plane C–H band. In Fig. S3b, the sharp absorption bond at 580 cm^−1^ was ascribed to the tetrahedral structure of the Fe–O bond. Figure S3c shows that by adding silver nanoparticles to the surface of the biochar–Fe_3_O_4_ substrate, there was no considerable change in the spectrum. Therefore, it can be concluded that the biochar–Fe_3_O_4_ substrate was stable during the synthesis of silver (Ag) nanoparticles.

### X-ray diffraction (XRD)

In Fig. S4, the X-ray diffraction patterns of the silver (Ag) nanoparticles synthesized from the extract are shown. The peaks were detected at 2θ values of 77.31°, 64.5°, 57.47°, 54.98°, 46.4°, 44.26°, 38.13°, 32.22°, and 27.92°, which are related to the (311), (220), (241), (142), (231), (200), (111), (122) and (210) silver crystalline planes, with face-centered cubic structures (JCPDS, file No. 04-0783). The silver nanoparticles synthesized by the extract have a crystal nature that is observable according to the XRD results. The average crystallite size of silver (Ag) nanoparticles was measured by applying Debye–Scherer’s equation. The size of the achieved Ag nanoparticles was calculated at 15.2 nm from the breadth of the plane (122) reflection.

The XRD analysis of the biochar/Fe_3_O_4_–Ag nanocomposite is exhibited in Fig. S5. In this pattern, a characteristic peak at 22.8° was observed, which was related to the reflection of the (002) crystal plane of the carbon-based biochar substrate. The diffraction peaks at 2Ɵ = 62.84°, 57.22°, 53.68°, 43.26°, 35.59°, and 30.22° were ascribed to the reflection planes of (440), (511), (422), (400), (311) and (220) for Fe_3_O_4,_ respectively. These patterns confirmed the face-centered cubic structure of Fe_3_O_4_ nanoparticles (JCPDS card no. 19‐0629). Additionally, several Bragg reflection peaks were observed at 32.09°, 38.18°, 44.38°, 64.57°, and 77.56°, which can be ascribed to the reflections of the (122), (111), (200), (220), and (311) crystalline phases of the fcc-structured Ag nanoparticles, respectively (JCPDS card no. 65‐2871). Using Debye–Scherer’s equation, the crystallite size of nanoparticles was calculated to be 13.4 nm for Fe_3_O_4_ from the width of the plane (311) and 19.8 nm for Ag from the width of the plane (111).

### UV–Vis analysis of silver (Ag) nanoparticles

**Figure 1:**
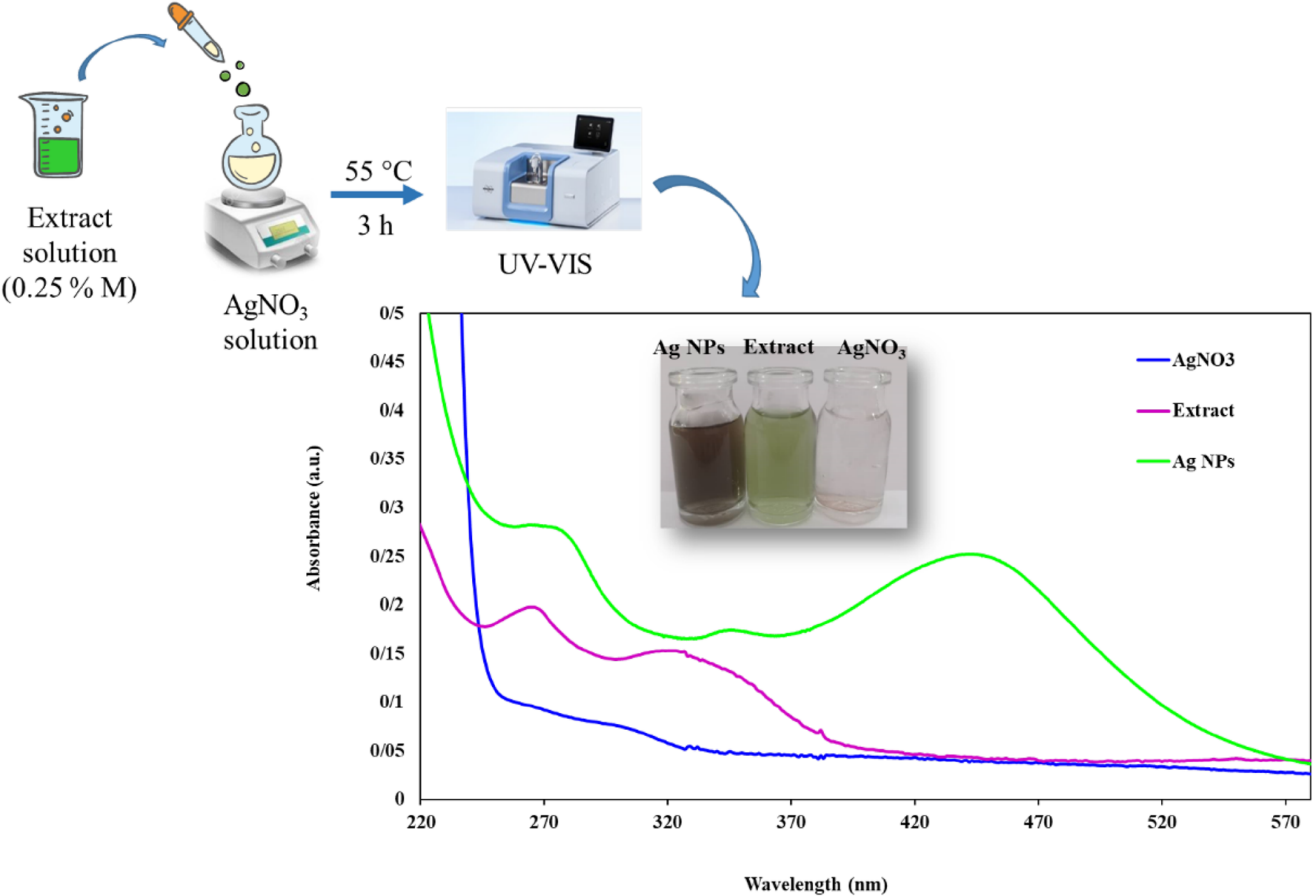
UV–Vis analysis of Celery leaf extract, pure AgNO_3_, and synthesized Ag nanoparticles.

UV–Vis analysis of leaf extract, silver nitrate (AgNO_3),_ and synthesized silver NP spectra are shown in Fig. 1. In this process, Ag nanoparticles were prepared using Celery leaf extract. Reducing Ag^+^ into Ag^0^ was confirmed by changing the color of the reaction mixture from light green to brown. As shown in Fig. 1, the leaf extract and AgNO_3_ solution did not show any absorbance peak in the range of 400–800 nm, but by adding the leaf extract into the AgNO_3_ solution, good absorbance was observed at 440 nm, which was relevant to the SPR (surface plasmon resonance) of silver (Ag) nanoparticles.

### FESEM and TEM analysis

**Figure 2:**
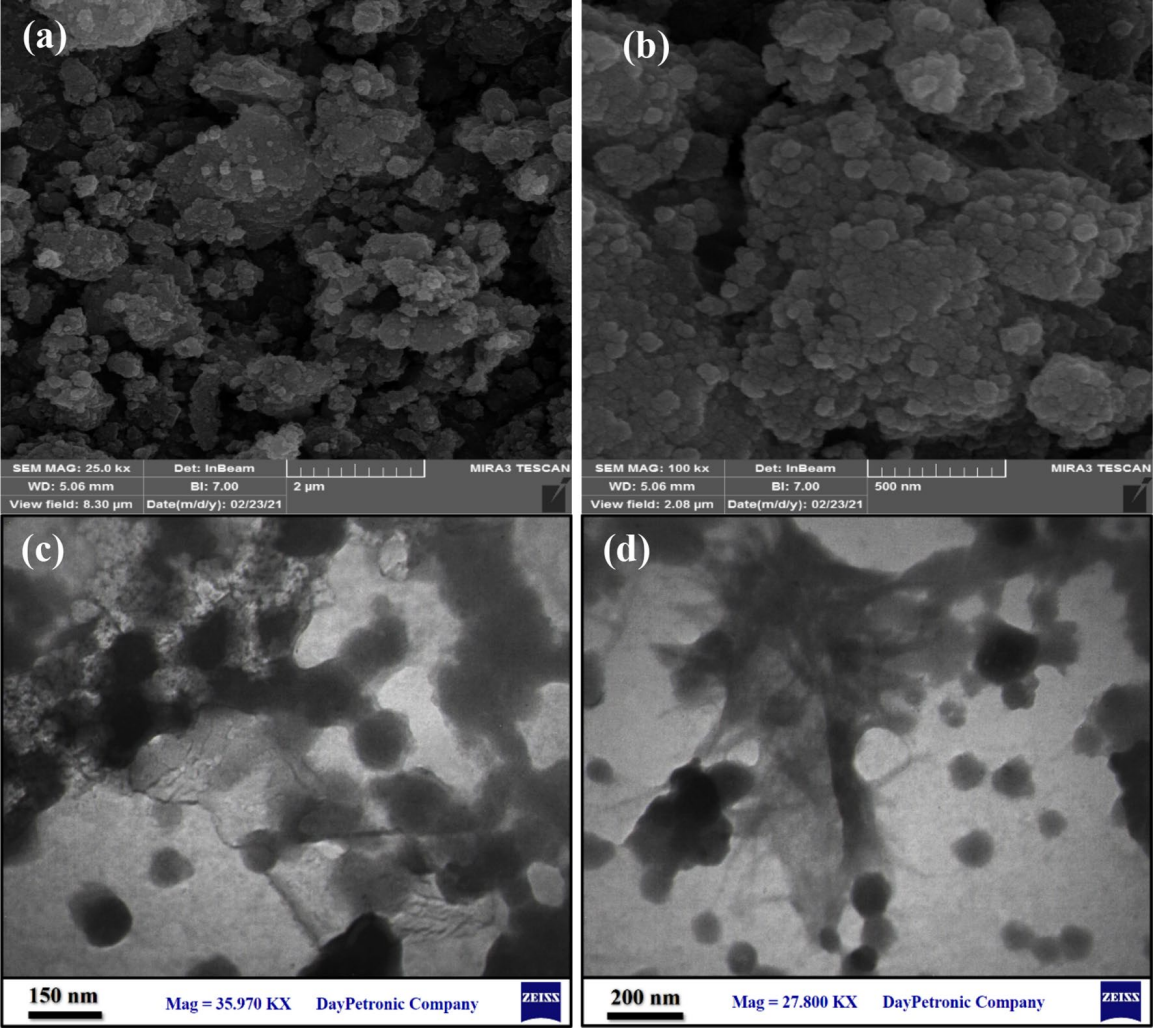
(**a**, **b**) FESEM images and (**c**, **d**) TEM images of the biochar/Fe_3_O_4_–Ag nanocomposite.

The FESEM (field emission scanning electron microscopy) images of the biochar/Fe_3_O_4_–Ag nanocatalyst in the range of 500 nm–2 µm are shown in Fig. 2a and b, which illustrates that the nanocatalyst consists of Fe_3_O_4_ and Ag spherical nanoparticles. It was also observed that the approximate size of the nanoparticles was 45–50 nm. FESEM images of biochar/Fe_3_O_4_–Ag show that the surfaces of biochar are well decorated with Ag and Fe_3_O_4_ nanoparticles. Figure 2c and d represents the TEM images of the obtained nanocomposite. The TEM images indicate the distribution of Fe_3_O_4_ and Ag nanoparticles with a size range of 40–50 nm on the biochar substrate. The size and morphology of Ag nanoparticles depended on the green extract used for synthesis.

### Energy dispersive spectrometry (EDS)-mapping

**Figure 3:**
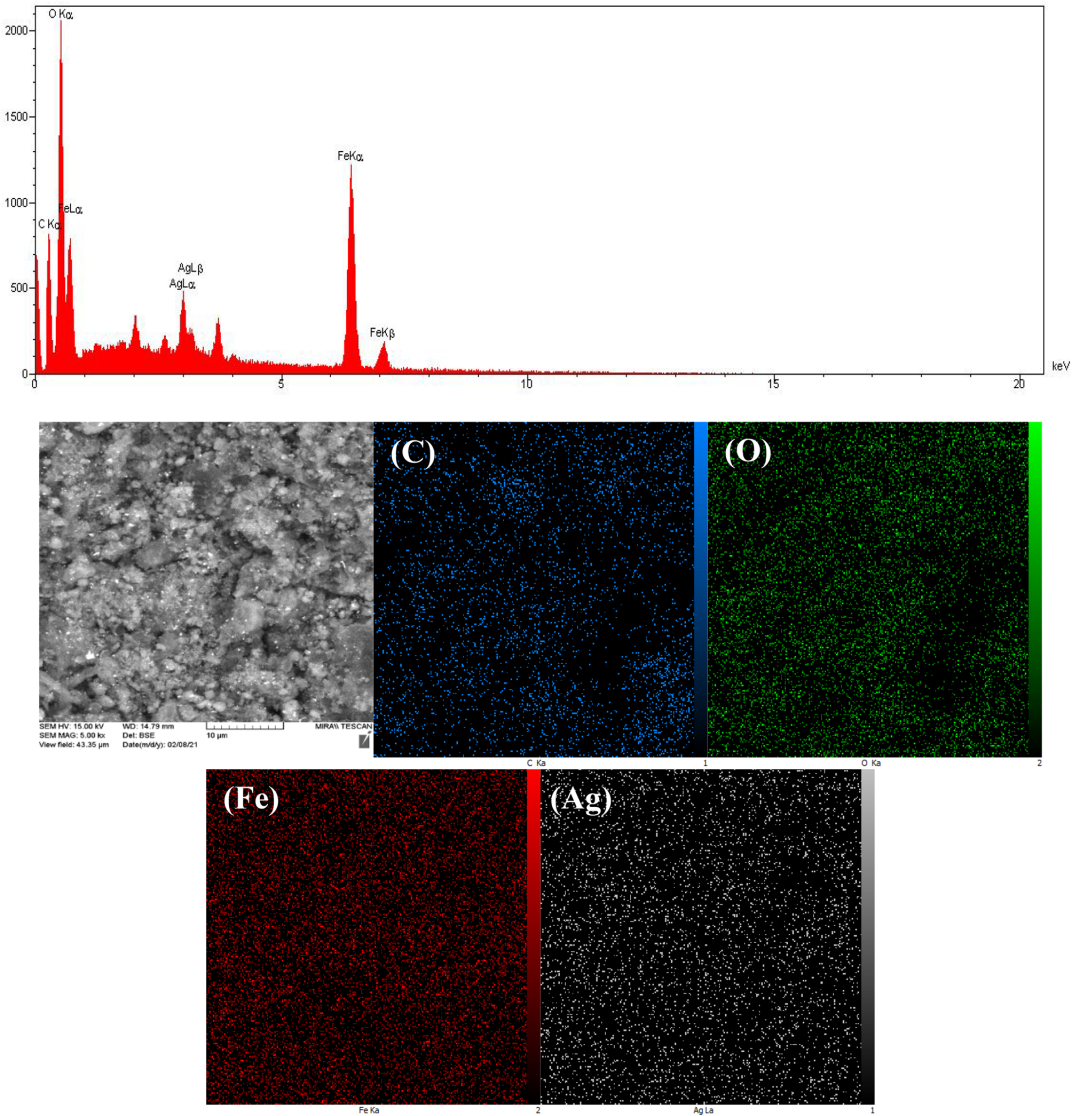
EDX-mapping analysis of biochar/Fe_3_O_4_–Ag nanocomposite.

To further investigate the structure of the biochar/Fe_3_O_4_–Ag nanocomposite, EDS mapping analysis was used to detect the distribution of the elements on the surface of the nanocomposite. Figure 3 represents the EDS mapping images of this nanocomposite. In light of the outcomes, the presence of the C, O, Fe, and Ag elements in the structure of the biochar/Fe_3_O_4_–Ag nanocatalyst was confirmed, which confirms the consolidation of Fe_3_O_4_ and Ag nanoparticles on the carbon-based biochar substrate.

### Vibration sampling magnetometer (VSM) analysis

**Figure 4:**
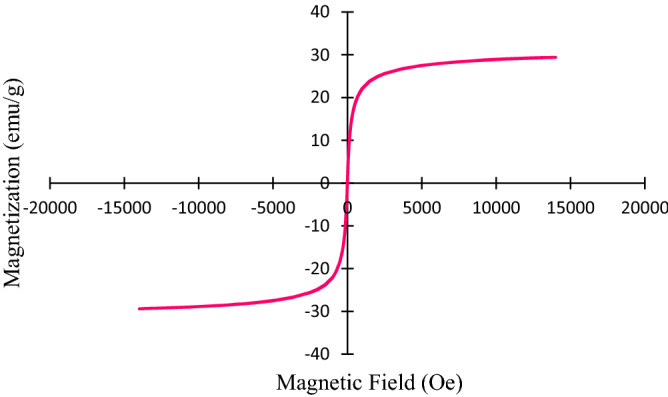
Magnetization curve of biochar/Fe_3_O_4_–Ag nanocomposite.

The appraisal of the magnetic properties of the biochar/Fe_3_O_4_–Ag nanocomposite was carried out utilizing the VSM technique. The VSM chart of the biochar/Fe_3_O_4_–Ag nanocomposite is shown in Fig. 4. According to the curve obtained from the VSM, biochar/Fe_3_O_4_–Ag has magnetic properties, and its saturation magnetization value was 29.4 emu/g. Additionally, due to the absence of a hysteresis loop, this nanocomposite has superparamagnetic properties. This magnetic behavior of the prepared nanocatalyst causes the particles to accumulate rapidly in the attendance of an external magnet, and the particles are easily dispersed as soon as the external magnet is removed.

### Catalytic performance

#### The nitroaromatic compounds reduction reaction

The reduction of 4-nitroaniline (0.5 mmol) was considered the model reaction to optimize the reduction reaction conditions of nitroaromatics. The quantity of biochar/Fe_3_O_4_–Ag nanocatalyst, temperature, and type of solvent were changed and evaluated to achieve the optimized value, as shown in Table S1.

To optimize the reaction conditions, first, different amounts of catalyst were examined. The results showed that in the absence of the catalyst, the reduction reaction did not occur (Table S1, Entry 1). Therefore, the presence of biochar/Fe_3_O_4_–Ag nanocatalysts is a vital factor for the reduction reaction. As seen, 10 mg of catalyst was considered a reasonable and optimal value (Table S1, Entry 4). Additionally, based on the results, it was found that by increasing the amount of nanocatalyst, the reaction time was decreased, and the yield was increased (Table S1, Entries 2–5).

After determining the optimal value of the nanocatalyst, the efficacy of temperature on the progress of the reduction reaction was surveyed. Based on the outcomes, it was observed that increasing the temperature led to higher performance and yield of the reaction as well as decreasing the reaction time (Table S1, Entries 6, 7). Therefore, due to less energy consumption, 50 °C was selected as the appropriate and optimal temperature for this reaction (Table S1, Entry 4).

Finally, to investigate the effect of the solvent, the model reaction was performed in the presence of different solvents (Table S1, Entries 8–13). Following the outcomes, H_2_O, as a green, cheap and stable solvent, indicated the best performance with a 98% yield (Table S1, Entry 4). These results illustrate that biochar/Fe_3_O_4_–Ag has good catalytic efficiency for the reduction of nitroaromatic compounds by utilization of NaBH_4_ as the reducing agent in water.

Following the results of the optimization experiments, the optimal conditions for the reduction reaction of nitroaromatic compounds were 10 mg of the biochar/Fe_3_O_4_–Ag synthesized nanocatalyst in water at 50 °C. After obtaining the optimized conditions, the reduction reactions of different nitroaromatic compounds under these conditions were investigated, and the results are illustrated in Table S2. The first compound, 4-nitroaniline, was reduced at a yield of 98% in 60 min (Table S2, Entry 1). Substituted nitroaromatic compounds such as amine, acid, and hydroxyl-nitrobenzenes were also reduced with high reaction performance and a yield of more than 95% in the reaction time range of 40–80 min (Table S2, Entries 2–8).

#### Reusability of the biochar/Fe_3_O_4_–Ag nanocatalyst for the nitroaromatic reduction reaction

**Figure 5:**
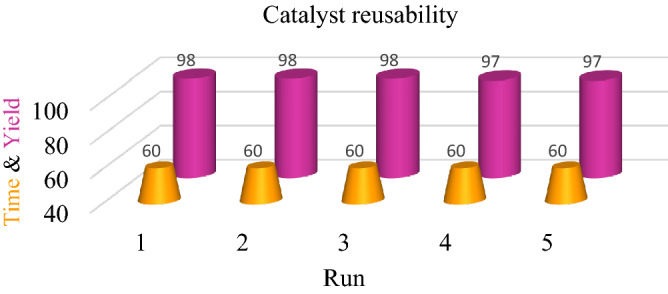
Reusability of the biochar/Fe_3_O_4_–Ag nanocatalyst for the nitroaromatic reduction reaction.

Recyclability is an important factor to evaluate a catalyst. Therefore, recycling experiments were accomplished to appraise the stability and activity of the catalyst. Under the optimized conditions, the biochar/Fe_3_O_4_–Ag nanocatalyst was separated by an external magnet after the nitroaromatic compound reduction reaction was completed, and then it was washed, dried, and used for subsequent cycles. The recovered catalyst was reused up to 5 times without substantial reduction in catalytic activity, and the results are demonstrated in Fig. 5. These results demonstrated that the biochar/Fe_3_O_4_–Ag catalyst has privileged properties, such as good catalytic performance, cost-effectiveness, facile and green synthesis, good stability, and recyclability, which made it an adequate catalyst for the reduction of nitroaromatic compounds.

#### Aldehyde and ketone compounds reduction reaction

To optimize the reduction reaction conditions of aldehydes and ketones, such as the amount of biochar/Fe_3_O_4_–Ag nanocatalyst, temperature, type of solvent, and the amount of NaBH_4_ as a reducing agent, the reduction of benzaldehyde (0.5 mmol) as a model reaction was examined, and it is demonstrated in Table 1.

**Table 1:** The effect of biochar/Fe_3_O_4_–Ag nanocatalyst amount, temperature, solvent, and NaBH_4_.


Entry	Catalyst (mg)	T (°C)	Solvent	NaBH_4_ (mmol)	Time (min)	Yield (%)^a^
1	–	25	H_2_O	1	40	97
2	2	25	H_2_O	1	12	97
3	3	25	H_2_O	1	10	97
**4**	**5**	**25**	H_2_O	**1**	**3**	**98**
5	5	50	H_2_O	1	2	98
6	5	25	Ethanol	1	13	60
7	5	25	H2O:ethanol (1:1)	1	5	85
8	5	25	Acetonitrile	1	6	87
9	5	25	THF	1	8	96
10	5	25	DMSO	1	6	45
11	5	25	DMF	1	9	75
12	5	25	CHCl_3_	1	30	trace
13	5	25	H_2_O	0.5	10	95
14	5	25	H_2_O	2	1	98

Significant values are in bold.

Reaction condition: benzaldehyde (0.5 mmol), solvent (2 mL).

^a^Isolated yield.

First, the effect of the biochar/Fe_3_O_4_–Ag amount was determined by keeping the other reaction conditions firm. In the absence of the catalyst, the reduction reaction time was long (Table 1, Entry 1). Additionally, according to the results, it was found that by adding the amount of biochar/Fe_3_O_4_–Ag nanocatalyst, the reaction time decreased, and the yield increased (Table 1, Entries 2–4). Therefore, 5 mg of biochar/Fe_3_O_4_–Ag was selected as the optimal value because of the short reaction time and high yield (Table 1, Entry 4).

After opting for the appropriate amount of catalyst, the efficacy of temperature on the reaction progress was determined. Depending on the results (Table 1, Entry 4), 25 °C was determined to be the optimal temperature for this reaction because this temperature complies with the laws of green chemistry. Additionally, it was observed that increasing the temperature led to a decrease in the reaction time (Table 1, Entry 5).

After determining the appropriate temperature, the next step is to peruse the effect of the solvent on the attendance of the biochar/Fe_3_O_4_–Ag catalyst in the reaction progress. As a function of the outcomes, H_2_O with a yield of 98% (Table 1, Entry 4) and THF with a yield of 96% (Table 1, Entry 9) were suitable solvents for this reaction, but H_2_O was selected as the optimal solvent due to its green, inexpensive and high yield. Additionally, other solvents, such as ethanol, acetonitrile, and DMF, had a yield between 60 and 87% (Table 1, Entries 6, 8, 11).

Finally, NaBH_4_ was used as a reducing agent, and its different concentrations were investigated to assess the optimum value while maintaining that the other parameters were constant. With the enhancement of the concentration of NaBH_4_, the yield was enhanced (Table 1, Entries 13, 14). Therefore, the concentration of 1 mmol NaBH_4_ was determined as the optimized value (Table 1, Entry 4). These results elucidate that biochar/Fe_3_O_4_–Ag is an outstanding catalyst for the reduction of aldehyde and ketone compounds in the presence of NaBH_4_ as the reducing agent in water.

As a first example, benzaldehyde was reduced at a yield of 98% in 3 min (Table 2, Entry 1). Substituted aldehyde and ketone compounds were also reduced with great reaction performance; the yield was more than 85% in the reaction time range from 3 to 10 min for aldehydes, and the yield was more than 30% in the reaction time range from 6 to 60 min for ketones.

**Table 2:** The reduction reaction of aldehyde and ketone compounds in the presence of biochar/Fe_3_O_4_–Ag nanocatalyst.

Entry	Aldehyde/ketone	Product	Time (min)	Yield (%)^a^
1			3	98
2			4	98
3			5	98
4			7	97
5			6	97
6			10	95
7			10	85
8			10	90
9			6	80
10			45	60
11			60	30

Reaction condition: aldehyde and ketone compounds (0.5 mmol), catalyst (5 mg), H_2_O (2 mL), NaBH_4_ (1 mmol), RT.

^a^Isolated yield.

For sample, IR spectra were taken from six derivatives, the peaks of which are as follows:

- *p-Aminophenol* functional groups of C–N (1255 cm^−1^), C=C (1512 cm^−1^), N–H bending (1614 cm^−1^), as well as N–H stretching, and O–H (3442–3278 cm^−1^).
- *4-Aminoacetophenon* functional groups of C–N (1253 cm^−1^), C=C (1517 cm^−1^), N–H bending (1615 cm^−1^), C=O (1726 cm^−1^), and N–H stretching (3391 cm^−1^).
- *2-Aminoaniline* functional groups of C–N (1272 cm^−1^), C=C (1498 cm^−1^), N–H bending (1633 cm^−1^), and N–H stretching (3429–3292 cm^−1^).
- *1,3-Diaminobenzene* functional groups of C–N (1323 cm^−1^), C=C (1494 cm^−1^), N–H bending (1604 cm^−1^), and N–H stretching (3421–3211 cm^−1^).
- *4-Aminoaniline* functional groups of C–N (1261 cm^−1^), C=C (1515 cm^−1^), N–H bending (1627 cm^−1^), and N–H stretching (3452–3369 cm^−1^).
- *3-Aminbenzoic acid* functional groups of C–N (1348 cm^−1^), C=C (1533 cm^−1^), N–H bending (1614 cm^−1^), C=O (1712 cm^−1^), and N–H stretching and O–H (3383 cm^−1^).

The IR spectra of these derivatives are shown in Fig. S6.

#### Reusability of the biochar/Fe_3_O_4_–Ag nanocatalyst for aldehyde and ketone reduction reactions

**Figure 6:**
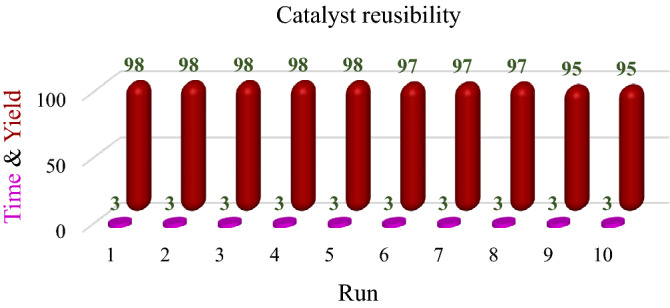
Reusability of the biochar/Fe_3_O_4_–Ag nanocatalyst for aldehydes and ketones reduction reaction.

Catalyst recovery and reusability are fundamental factors in the nomination of the efficiency and performance of the catalyst. In this regard, the recoverability of the biochar/Fe_3_O_4_–Ag nanocatalyst was investigated in the model reaction of the reduction of aldehydes and ketones under optimal conditions. The results of the performance testing demonstrated that the synthesized nanocatalyst could be applied in at least 10 successive runs without a substantial reduction in catalytic performance, which affirmed the heterogeneous nature of the catalyst. The results are shown in Fig. 6. Based on the outcomes, good catalytic performance, excellent stability, and recoverability, biochar/Fe_3_O_4_–Ag is an appropriate catalyst for the reduction of aldehydes and ketones.

## Conclusion

This study was centralized on the green synthesis of biochar as a carbon-based substrate and Ag nanoparticles using leaf extract of Celery. In general, a fast and ecofriendly synthesis process for silver nanoparticles in the presence of a green precursor and solvent has been illustrated. The structural, morphological, and optical properties of the Ag nanoparticles were determined by diverse techniques. The prepared biochar/Fe_3_O_4_–Ag nanocatalyst exhibited excellent catalytic efficiency for the reduction of nitroaromatic compounds, aldehydes, and ketones in the presence of NaBH_4_ as a reducing agent and H_2_O as a green solvent and possessed appropriate reusability. The advantages of this heterogeneous catalyst include green conditions such as low reaction temperature, green solvent, short reaction time, easy separation, low cost, and eco-friendliness. The biochar/Fe_3_O_4_–Ag nanocatalyst could be segregated by the utilization of an external magnet and reused five to ten times without appreciable loss of its catalytic performance.

## Supplementary Information

Supplementary Information.
