# Supplementary material for: Synthesis of Ag nanoparticles by Celery leaves extract supported on magnetic biochar substrate, as a catalyst for the reduction reactions
Source: Sci Rep. 2022 Aug 11;12:13678. doi: 10.1038/s41598-022-18131-w (PMC9372062; doi:10.1038/s41598-022-18131-w)
Supplement: Supplementary file 1 — Supplementary Information. [file 41598_2022_18131_MOESM1_ESM.docx]

Figure S1. Scheme of the process for preparing *Celery* leaf extract

**
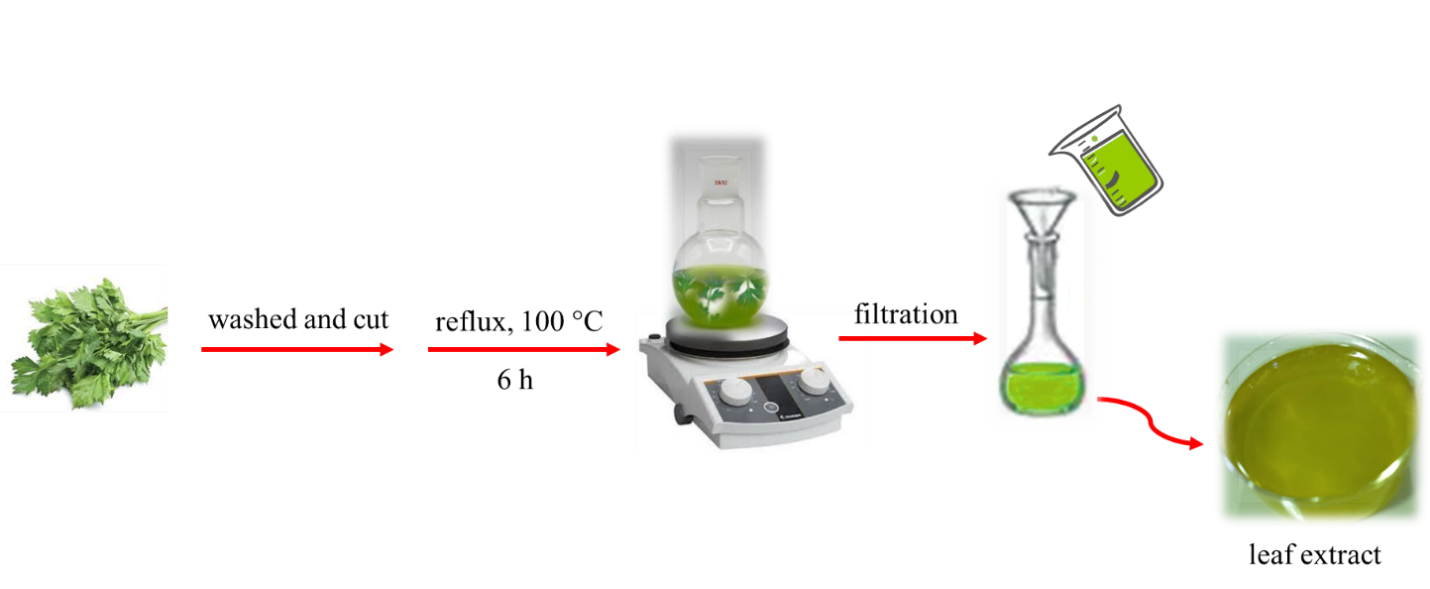
**

Figure S2. The process of Biochar/Fe_3_O_4_-Ag nanocatalyst synthesis


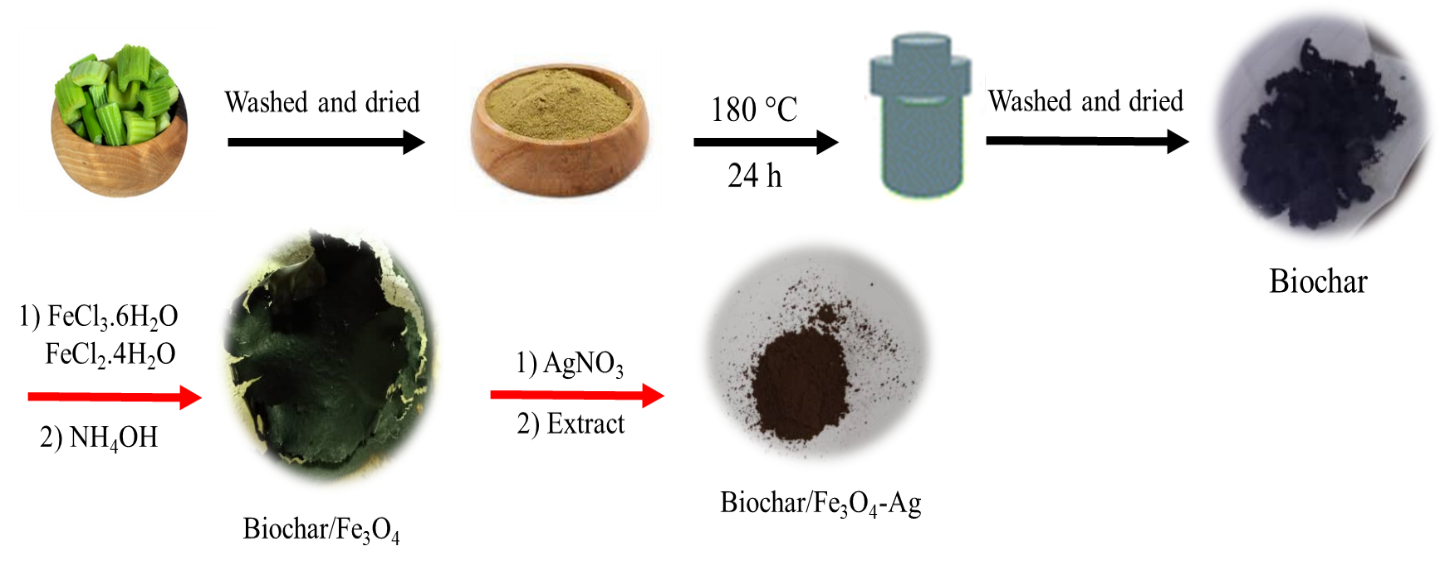


Figure S3. FT-IR spectra of (a) Biochar, (b) Biochar-Fe_3_O_4,_ and (c) Biochar/Fe_3_O_4_-Ag


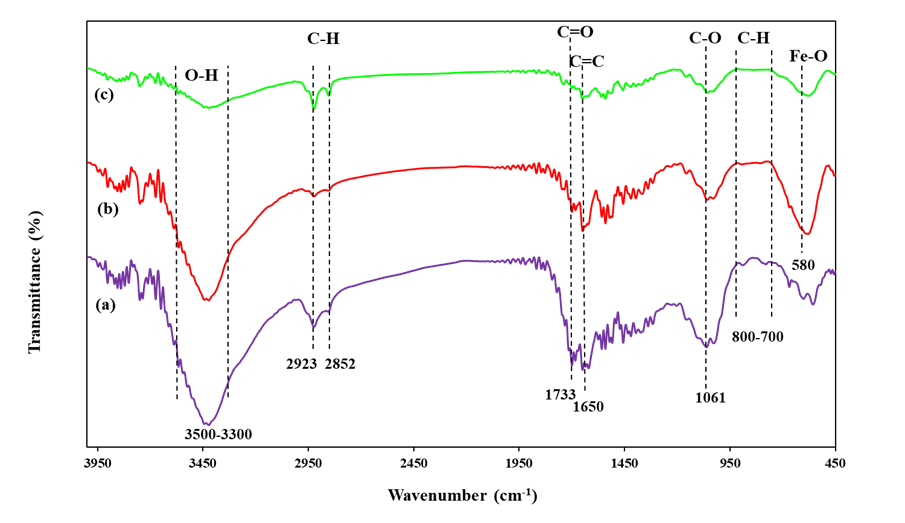


Figure S4. XRD pattern of synthesized Ag nanoparticles by the extract


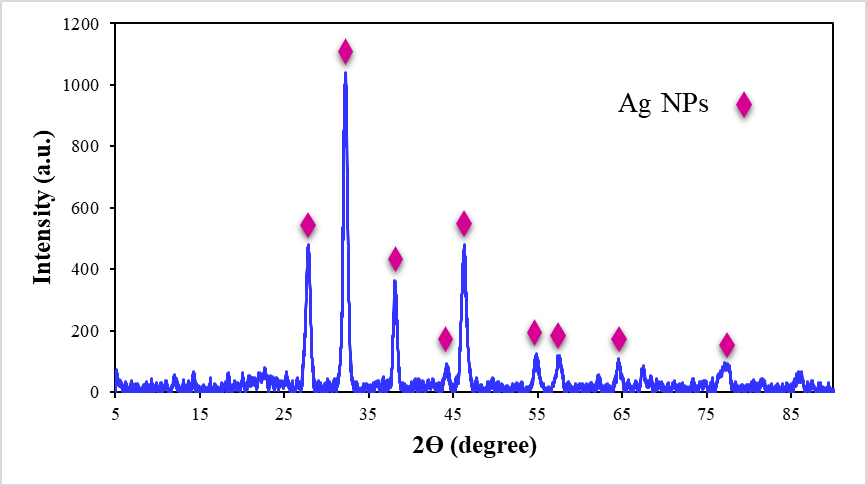


Figure S5. XRD pattern of Biochar/Fe_3_O_4_-Ag nanocomposite


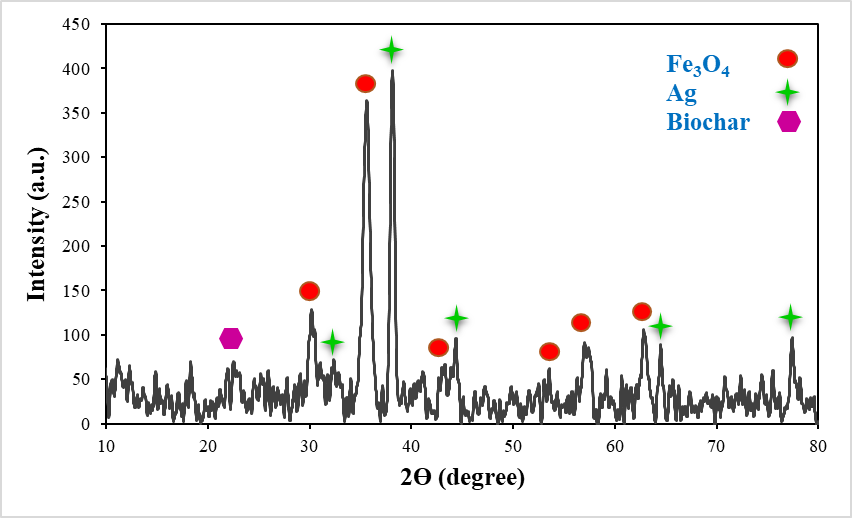


Figure S6. FTIR spectra of (a) p-Aminophenol, (b) 4-Aminoacetophenon, (c) 2-Aminoaniline, (d) 1,3-Diaminobenzene, (e) 4-Aminoaniline, and (f) 3-Aminbenzoic acid

Table S1. The effect of Biochar/Fe_3_O_4_-Ag nanocatalyst amount, Temperature, and Solvent **^a^**

|  | | | | | |
| --- | --- | --- | --- | --- | --- |
| Entry | Catalyst (mg) | T (°C) | Solvent | Time (min) | Yield (%)**^b^** |
| 1 | - | 50 | H_2_O | 180 | Trace |
| 2 | 5 | 50 | H_2_O | 60 | 55 |
| 3 | 8 | 50 | H_2_O | 60 | 95 |
| **4** | **10** | **50** | **H_2_O** | **60** | **98** |
| 5 | 20 | 50 | H_2_O | 50 | 98 |
| 6 | 10 | 25 | H_2_O | 50 | 35 |
| 7 | 10 | 75 | H_2_O | 20 | 98 |
| 8 | 10 | 50 | H_2_O: Ethanol (1:1) | 35 | 55 |
| 9 | 10 | 50 | Acetonitrile | 60 | Trace |
| 10 | 10 | 50 | Toluene | 60 | Trace |
| 11 | 10 | 50 | DMSO | 60 | 5 |
| 12 | 10 | 50 | DMF | 60 | Trace |
| 13 | 10 | 50 | CHCl_3_ | 60 | Trace |
| **^a^** Reaction Condition: 4-Nitroaniline (0.5 mmol), Solvent (3 mL), NaBH_4_ (3 mmol)  **^b^** Isolated yield | | | | | |

Table S2. The reduction reaction of Nitroaromatics in the presence of Biochar/Fe_3_O_4_-Ag nanocatalyst **^a^**

|  | | | | | |
| --- | --- | --- | --- | --- | --- |
| Entry | Nitroaromatic | Product | Time (min) | Yield (%)**^b^** | Melting point /m.p.Lit (°C) [67] |
| 1 |  |  | 60 | 98 | 144/145-147 |
| 2 |  |  | 80 | 75 | 101-103/102-104 |
| 3 |  |  | 50 | 96 | 176-178/178-180 |
| 4 |  |  | 40 | 95 | 144-146/146-148 |
| 5 |  |  | 45 | 97 | 187-189/187-190 |
| 6 |  |  | 50 | 97 | 170-172/170-174 |
| 7 |  |  | 70 | 95 | Oil/-6 |
| 8 |  |  | 65 | 96 | 63-65/64-66 |
| **^a^** Reaction Condition: Nitro compounds (0.5 mmol), Catalyst (10 mg), H_2_O (3 mL), NaBH_4_ (3 mmol), 50°C  **^b^** Isolated yield | | | | | |
